# Reconstruction using the colon or jejunum in patients with synchronous advanced esophageal and gastric cancers: a retrospective study from a single institutional database

**DOI:** 10.1186/s12893-023-02072-w

**Published:** 2023-06-27

**Authors:** Rongrong Jiang, Youbo Wang, Juefeng Xu, Zhiming Chen, Liewen Pang

**Affiliations:** 1 Department of Cardiothoracic Surgery, Huashan Hospital, Fudan University, #12 Wulumuqi Rd. (M), Shanghai, 200040 P.R. China; 2 Department of Nursing, Huashan Hospital, Fudan University, Shanghai, P.R. China

**Keywords:** Synchronous tumors, Esophageal squamous cell carcinoma, Gastric cancer, Reconstruction

## Abstract

**Purpose:**

The aim of this study was to evaluate the feasibility and efficacy of simultaneous resection of synchronous advanced esophageal and gastric cancers.

**Methods:**

We retrospectively analyzed the clinical data of 16 patients who underwent resection of synchronous advanced esophageal squamous cell carcinoma (ESCC) and gastric adenocarcinoma from January 2009 to Dec 2021. Subtotal esophagectomy and total gastrectomy were performed using the Ivor-Lewis or McKeown approach. Reconstruction was performed using a pedicled jejunal graft or colon interposition. Perioperative and postoperative data of all patients were analyzed.

**Results:**

There were no in-hospital mortalities following surgery, but 9 patients (56.3%) suffered major perioperative complications. Comparison of the groups that received reconstruction using the jejunum and the colon indicated similar incidences of perioperative complications, overall survival, and disease-free survival. Cox regression analysis indicated that lymph node metastasis of both cancers was independent risk factor for overall survival.

**Conclusion:**

The existence of synchronous tumors of the esophagus and stomach is not unusual, the radical surgical treatment could be carried out whenever possible.

## Introduction

A recent review of cancer statistics in China reported that gastric cancer was the second most common cancer and esophageal cancer was the sixth most common [1]. Moreover, these two cancers are responsible for about half of all cancer mortalities worldwide [2]. Many of the risk factors are shared in these two cancers, especially the use of alcohol and tobacco, and poor social and economic status. Synchronous or metachronous occurrence of gastric cancer can occur in patients with esophageal cancer [3, 4]. In particular, Ito et al. reported that about 5.3% of patients with esophageal cancer had synchronous gastric cancer [5], and the nationwide registry of Japan reported that about 4.3% of patients with esophageal cancer had synchronous gastric cancer [6].

Thus, surgeons may face the dilemma of patients presenting with simultaneous gastric and esophageal cancers in clinical settings, but there are no established treatment guidelines for these patients. Reconstruction of the continuity of the alimentary tract using the colon or jejunum can be difficult in patients receiving synchronous esophagogastrectomy. Due to high morbidity and mortality associated with surgery, even in high-volume referral centers, many doctors and patients would select palliative care, such as chemotherapy, radiotherapy, or chemoradiotherapy [7, 8].

To our knowledge, there are currently few literature reports on the synchronous esophagogastrectomy for this rare disease. In this study, we retrospectively analyzed the clinical data of 16 patients who received synchronous resection of advanced esophageal squamous cell carcinoma (ESCC) and gastric adenocarcinoma at our institution, and evaluated the feasibility and efficacy of this treatment modality and analyzed the impacts of different clinical parameters on the overall survival.

## Patients and methods

### Patients

This study was performed in accordance with the Declaration of Helsinki and was approved by Ethics Committee of Huashan Hospital, an affiliate of Fudan University (Shanghai). Because this was a retrospective study, the need for informed consent was waived, according to Ethics Committee of Huashan Hospital.

From January 2009 to December 2021, patients who had primary ESCC and gastric adenocarcinoma and received simultaneous radical surgical resection at the Department of Cardiothoracic Surgery in Huashan Hospital were retrospectively enrolled. Synchronous gastric cancer was defined as detection of gastric cancer before surgery based on preoperative esophagogastroduodenoscopy(EGD) that was pathologically proven as adenocarcinoma.Any patient who received simultaneous endoscopic resection for esophageal or gastric cancer was excluded. Patients with early tumors located in the upper gastric cancer and adenocarcinoma infiltrating the dentate line were also excluded. Patients in poor condition or with severe cardiopulmonary dysfunction were excluded. At the time of diagnosis, each patient underwent thorough staging workup, including EGD, axial imaging with computed tomography (CT), and typically positron emission tomography (PET), in accordance with guidelines. A multi-disciplinary team discussed each patient before surgery.

### Surgical procedures

Prior to surgery, different operation schemes were devised according to the location, extension, and size of the tumors. In all patients, subtotal esophagectomy and total gastrectomy using the Ivor- Lewis or the McKeown approach were performed, with reconstruction using a pedicled jejunal graft or colon interposition. In general, patients with tumors in the proximal or mid-thoracic esophagus received the McKeown approach, and those with distal tumors received the Ivor-Lewis approach. Cervical node dissection was performed for upper esophageal tumors and when there was upper mediastinal node metastasis. Cervical or intrathoracic anastomosis was performed using a 25- or 28-mm circular stapling gun. Additional microvascular augmentation technique was not employed.

### Esophageal reconstruction using a pedicled jejunal graft

When Roux-en-Y reconstruction with anastomosis between the esophagus and jejunum was performed using a pedicled jejunal graft, a cut of the second or third jejunal vessels was used to elongate the graft.

### Esophageal reconstruction with colon interposition

Two main types of colonic graft were used for esophageal reconstruction. The segment from the terminal ileum to the ascending colon was interposed isoperistaltically using the middle colic vessels as a pedicle, while dividing the right colic and ileocolic vessels. The other type was that the segment from the transverse colon to the splenic flexure was used for interposition isoperistaltically, using the ascending branch of the left colic artery and the inferior mesenteric vein as a pedicle, while dividing the middle colic vessels.

### Outcomes and follow-up

The composite short-term outcomes of major postoperative morbidity or mortality was defined as a major pulmonary complication (acute respiratory distress syndrome, pneumonia and respiratory failure requiring reintubation, or tracheostomy), a major cardiovascular complication (arrhythmia requiring pharmacologic intervention, myocardial infarction, pulmonary embolism, or cardiac arrest), anastomotic leak (requiring endoscopic intervention of grade II or greater), chylothorax requiring operative intervention, and death within 90 days after the operation [9, 10].

Postoperatively, patients were typically followed with clinical examination and CT or PET/CT every 6 months for two years, and then annually thereafter. Surveillance EGD was performed when indicated by clinical symptoms. When there were any signs and symptoms of cancer metastasis, the extra examinations would be performed.

The long-term outcomes included overall survival (OS) and disease-free survival (DFS). OS was defined as the time from completion of therapy to death from any cause. DFS was defined as the time from completion of therapy to tumor recurrence or death. Patients surviving at the end of the study period were censored at the date of the last follow-up.

### Statistical analysis

Statistical analysis was performed using SPSS version 23.0. All descriptive data were presented as median and interquartile range. Continuous data were compared using Student’s *t*-test and categorical data using the χ^2^ test. Survival curves were presented using the Kaplan-Meier method, and differences were assessed using the log-rank test. Multivariable analysis was performed using Cox regression. Three kinds of variables were included in the Cox regression. The variables which were significantly distribute in different Group J and C were adjusted, followed by the preoperative variables, then the postoperative variables. A two-sided P value below 0.05 was considered significant.

## Results

### Patient characteristics

From January 2009 to December 2021, 2125 patients at our institution received surgical resection for primary esophageal cancer, and 16 of these patients received synchronous surgical resection for double primary cancers of the esophagus and stomach (Table 1). In all cases, the esophageal carcinoma was detected first, and the gastric carcinoma was detected following detailed examination of the upper digestive tract. All patients received R0 resection for the esophageal and gastric cancers.

**Table 1:** Clinicopathologic characteristics of the two groups

**Characteristic**	Total	**Group J**	Group C	**t / χ** ^**2**^	p-value
	**(n = 16)**	(n = 5)	(n = 11)		
Age, Median(P25,P75) (years)	64(60,68)	60(51,64)	65(63,70)	2.632	0.020*
Male	14	5	9		1.000#
Hypertension	3	0	3		0.509#
Smoking	5	2	3		1.000#
Alcohol consumption	4	1	3		1.000#
Adjuvant Therapy					
None	4	3	1		
Preoperative	2	0	2		1.000#
Postoperative	10	2	8		1.000#
Location of esophageal-graft anastomosis					0.245#
Neck	4	4	0		
Chest	12	7	5		
**Esophageal cancer**					
Stage					1.000#
I	5	1	4		
II	0	0	0		
III	9	3	6		
IV	2	1	1		
Depth of tumor invasion					1.000#
pTis	1	0	1		
pT1	4	1	3		
pT2	2	1	1		
pT3	5	2	3		
pT4	4	1	3		
Lymph node metastasis					0.308#
Positive	9	4	5		
Negative	7	1	6		
Location					0.673#
Upper	1	0	1		
Middle	2	0	2		
Lower	13	5	8		
**Gastric cancer**					
Stage					0.547#
I	6	1	5		
II	4	1	3		
III	6	3	3		
IV	0	0	0		
Depth of tumor invasion					0.320#
pTis	4	1	3		
pT1	1	1	0		
pT2	3	0	3		
pT3	5	1	4		
pT4	3	2	1		
Lymph node metastasis					**0.036#**
Positive	6	4	2		
Negative	10	1	9		
Location					0.390#
Cardia	4	1	3		
Fundus	3	2	1		
Body	9	2	7		

J: reconstruction using the jejunum; C: reconstruction using the colon; *Student’s *t*-test; #Fisher’s exact test

The median patient age was 65 years (range, 45–73), and 14 patients were male. Histological examinations indicated the esophageal cancers were squamous cell carcinomas and the gastric cancers were adenocarcinomas. The esophageal cancers were at stage I (n = 5, 31.3%), III (n = 9, 56.3%), or IV (n = 2, 12.5%), and the gastric cancers were at stage I (n = 6, 37.5%), II (n = 4, 25%), or III (n = 6, 37.5%). One (6.3%) esophageal carcinoma was in the upper thoracic region, 2 (12.5%) were in the mid-thoracic region, and 13 (81.3%) were in the lower thoracic region. Four (25%) gastric tumors were located the cardia, 3 (18.8%) were in the fundus, and 9 (56.3%) were in the body. Among the esophageal tumors, 1 (6.3%) was pTis, 4 (25%) were pT1, 2 (12.5%) were pT2, 5 (31.3%) were pT3, and 4 (25%) were pT4. Among the gastric tumors, 4 (25%) were pTis, 1 (6.3%) was pT1, 3 (18.8%) were pT2, 5 (31.3%) were pT3, and 3(18.8%) were pT4. Lymph node metastases were found in 9 (56.3%) esophageal tumors and 6 (37.5%) gastric tumors.

### Operative data

Subtotal esophagectomy and total gastrectomy were performed with reconstruction using a pedicled jejunal graft (Group J, n = 5, 31.3%) or colon interposition (Group C, n = 11, 68.8%), using the Ivor-Lewis or McKeown approach (Table 2). In Group C, transthoracic esophagectomy with cervical anastomosis of the colonic conduit was performed in 4 patients, while intrathoracic anastomosis of the colonic conduit was performed in 7 patients; 9 patients received reconstruction *via* the posterior mediastinum and 2 *via* the retrosternal route. All 5 patients in Group J received esophagojejunostomy with intrathoracic anastomosis *via* the posterior mediastinum.

**Table 2:** Surgical techniques used in the two groups

Technique	Total (n = 16)	Group J (n = 5)	Group C (n = 11)	t / χ^2^	p-Value
Modus operandi					0.245#
Ivor-Lewis	12	5	7		
McKeown	4	0	4		
Route of reconstruction					1.000#
Posterior mediastinum	14	5	9		
Retrosternal	2	0	2		
Operative time, Median(P25,P75) (h)	7.71(6.92,9.42)	7.5(7.25,8.42)	7.92(6.83,9.83)	0.792	0.442*

J: reconstruction using the jejunum; C: reconstruction using the colon; *Student’s *t*-test; #Fisher’s exact test

### Outcomes

After resection, 9 patients (56.3%) experienced major complications, but there were no in-hospital moralities (Table 3). One patient experienced the potentially lethal complication of graft necrosis, requiring an emergency procedure. 3 patients, who received cervical anastomosis experienced anastomotic leakage, and 2 patients had symptomatic anastomotic stenosis requiring esophageal bougienage. Compared with the jejunal reconstruction group, the colon reconstruction group had similar perioperative outcomes, including anastomotic leakage, major pulmonary and cardiovascular complications, graft failure, and in-hospital mortality.

**Table 3:** Short-term outcomes of the two groups

Outcome	Total (n = 16)	Group J (n = 5)	Group C (n = 11)	p-Value
Major pulmonary complication	1	0	1	1.000#
Major cardiovascular complication	2	0	2	1.000#
Anastomotic leak	3	1	2	1.000#
Graft failure	1	1	0	0.312#
Anastomotic stenosis	2	1	1	1.000#
In-hospital mortality	0	0	0	-

#Fisher’s exact test

**Fig. 1:**
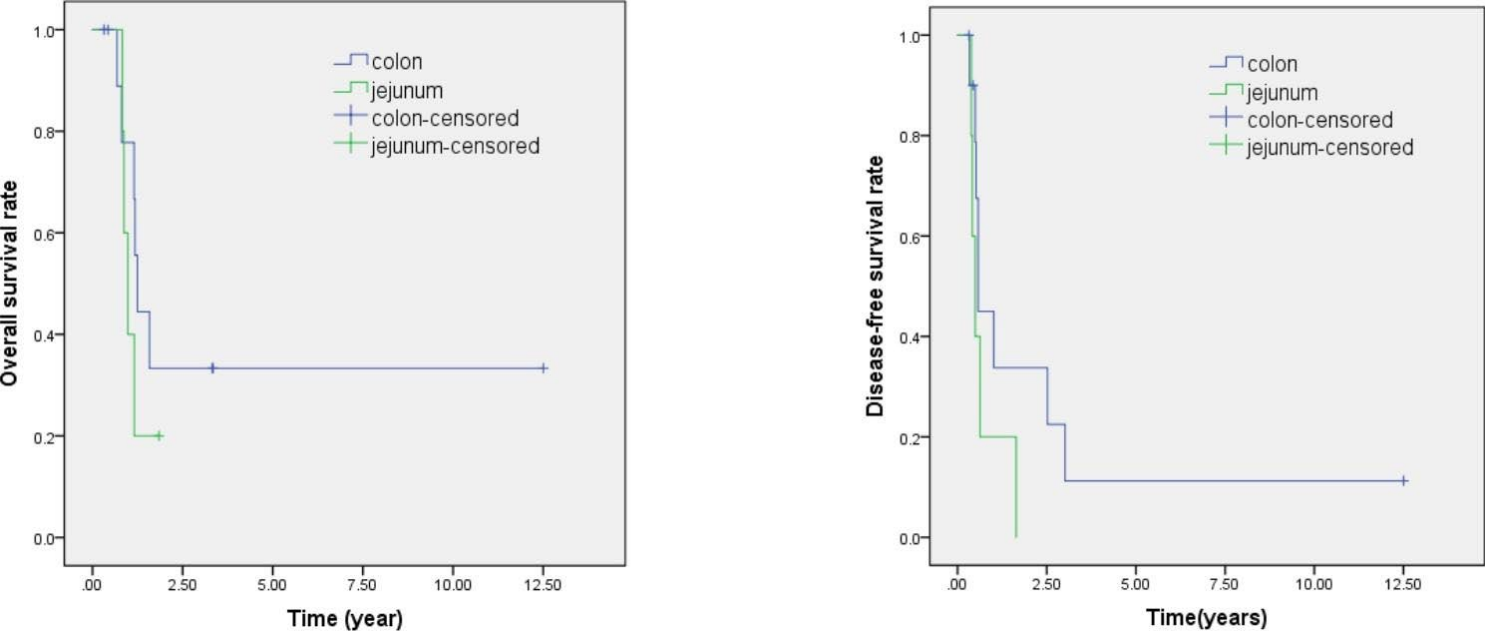
Overall survival (left) and disease-free survival (right) in patients who received reconstruction using the colon (blue) and using the jejunum (green). Log-rank tests indicated the two groups had no significant differences in median OS (colon: 1.244 years, jejunum: 0.981 years, P = 0.390) or median DFS (colon: 0.575 years, jejunum: 0.493 years, P = 0.177)

The median follow-up period was 13.9 months and no patients were lost to follow-up (Fig. 1). The median OS was 1.244 years in the Group C and 0.981 years in Group J (P = 0.390). The median DFS was 0.575 years in Group C and 0.493 years in Group J (P = 0.177). Log-rank tests indicated the two groups had no significant differences in median OS (colon: 1.244 years, jejunum: 0.981 years, P = 0.390) or median DFS (colon: 0.575 years, jejunum: 0.493 years, P = 0.177).

For the lymph node metastasis, we had conducted two analyses to test its effect. First, we test the difference of short-term outcomes in positive and negative lymph node metastasis subgroup (Table 4), but the results of χ2 test showed no significance, which means there was no difference in the distribution of patients with lymph node metastasis on the short-term outcomes. Secondly, for Cox regression results (Table 5), the stage of esophageal cancer, grades of esophageal and gastric cancer, reconstruction substitute, route of reconstruction and lymph node metastasis were all significant with OS.

**Table 4:** Short-term outcomes between positive and negative lymph node metastasis

Outcome	Lymph node metastasis		p-Value
	**Negative**	**Positive**	
Major pulmonary complication	1	0	1.000#
Major cardiovascular complication	1	1	1.000#
Anastomotic leak	2	1	1.000#
Graft failure	0	1	0.375#
Anastomotic stenosis	1	1	1.000#
In-hospital mortality	0	0	-

#Fisher’s exact test

**Table 5:** Cox regression analysis of factors associated with overall survival

Risk Factor	HR	p-value	95%CI
Age	1.767	0.709	0.089–35.172
Stage of esophageal cancer	3.672*10^6	0.019	11.710–1.151*10^12
Grades of esophageal cancer	0.000	0.022	0.000-0.188
Grades of gastric cancer	0.003	0.035	0.000-0.673
Modus operandi	81.446	0.130	0.275-24103.104
Reconstruction substitute	0.000	0.028	0.000-0.157
Route of reconstruction	1.005*10^7	0.019	14.171–7.125*10*12
Lymph node metastasis			
None	1 (ref)		
One cancer	1.206*10^13	0.013	557.181–2.612*10^23
Both cancers	2.817*10^19	0.013	11787.485–6.730*10^24

CI: confidence interval; HR: hazard ratio

Age was subgrouped by quartile: below 60 years old, 61–68 years old, older than 68

## Discussion

Some of the risk factors are shared in esophageal and gastric cancers, including high-fat diet [11], low socioeconomic status [12], alcohol consumption, and cigarette smoking [13]. Thus, it is not unusual for patients to present with synchronous gastric and esophageal cancers. Previous research reported that 5.3 to 6.1% of patients with thoracic esophageal carcinomas had synchronous gastric carcinomas [5, 14].

Surgical resection remains the cornerstone of the multimodal treatment for synchronous esophageal and gastric cancers, and the main objective of this surgery is reconstruction of the digestive tract. When a patient presents with synchronous esophageal and gastric cancers, there are three possible surgical modalities: endoscopic resection [15], partial resection of the stomach including proximal resection [16], and total gastrectomy. A 2013 study [12] reported that endoscopic submucosal dissection for gastric tumor, with Ivor-Lewis esophagectomy 1 to 2 weeks later, was an effective approach for patients with esophageal cancer and gastric epithelial neoplasia. Proximal gastrectomy is useful for patients who have early-stage gastric cancers in the upper third of the stomach. Y. Zhao [17] reported distal gastrectomy preserving the gastroepiploic vessels, Roux-en-Y gastrojejunostomy and thoracoscopic Ivor Lewis esophagectomy with chest anastomosis to deal with synchrouns esophageal tumor(located at least 27 cm away from the incisor teeth)and gastric tumor (located in the distal portion of the gastric tube and evaluated for clinical stage IA). The indication was so strict that the surgical plan could not popularize.

When a gastric adenocarcinoma is large (T2 or T3) or located in the distal region, the safety of partial gastrectomy may be questionable because it can be difficult for the surgeon to secure a safe margin, and extensive gastric resection might lead to an unsuitable blood supply to the gastric conduit. In such circumstances, total gastrectomy may be needed. Some surgeons may have concerns about the surgical burden caused by synchronous resection and the complicated reconstruction. We showed that there were no in-hospital mortalities, although 56.3% of patients suffered major complications after simultaneous resection of gastric and esophageal cancers. In addition, graft failure occurred in only one patient. These perioperative results enhanced our confidence in the surgical treatment of these complicated patients. Li et al. also reported that the perioperative and survival outcomes of patients with synchronous primary esophageal squamous and gastric cancers were not worse than those of patients with isolated esophageal cancer or isolated gastric cancer [18, 14].

After synchronous esophagectomy and total gastrectomy, the colon or pedicle jejunal can be used for reconstruction. In Japan, approximately 10% of patients receive esophageal reconstruction after oncologic esophagectomy using conduits other than the stomach [19]. However, the optimal conduit for patients who receive synchronous esophagogastrectomy is still uncertain. We recommend that patients who undergo synchronous esophagogastrectomy could receive reconstruction using a pedicled jejunal graft or colon interposition, using the Ivor-Lewis or McKeown approach according to the location, extension, and size of the tumors. In general, patients with tumors in the proximal or mid-thoracic esophagus received the McKeown approach, and those with distal tumors received the Ivor-Lewis approach. The pedicled jejunum is limited because the long segment of the jejunal loop is difficult to prepare and has poor connection with the marginal vessels; we therefore used it for lower anastomosis after partial resection of the lower esophagus. A major advantage of reconstruction using the colon is that a long graft is available. However, frequent variation in mesenteric vessels of the colon graft and the complicated surgical procedure, which requires three anastomoses rather than two (as in jejunal graft reconstruction) may prolong the operation time and increase intraoperative blood loss, possibly leading to increased mortality.

Although the operation time of our colonic reconstruction group was a little longer than our jejunum reconstruction group, the difference was no statistically significant. Moreover, compared with the jejunal reconstruction group, the colon reconstruction group had similar perioperative outcomes, including anastomotic leakage, major pulmonary and cardiovascular complications, graft failure, and in-hospital mortality. Doki et al. performed a retrospective study of esophageal cancer patients after gastrectomy and reported several benefits of jejunal reconstruction rather than colon reconstruction [15]. In particular, anastomotic leakage tended to be less frequent, the hospital stay was shorter, and the postoperative bodyweight loss was less in their jejunum reconstruction group [20]. However, it should be mentioned that in this study the surgeons employed supercharging and superdrainaging techniques in both reconstruction methods. However, these techniques were complicated and required the use of microsurgery, so it had not been widely adopted.

The median OS time in our study was 1.244 years for the colon reconstruction group and 0.981 years for the jejunum reconstruction group. Thus, compared with Li et al.[18], our patients had a slightly inferior OS. This may be explained that our patients had more advanced esophageal and gastric cancers (11 had phase III/IV esophageal cancer, 6 had phase III gastric cancer). Besides this, only a small portion of our patients received neoadjuvant chemotherapy, and none received preoperative or postoperative immunotherapy. The inferior OS may also be due to the synchronous multiple primary malignancy. Q. W. Li [8] reported that compared with matched non-multiple primary cancer, ESCC accompanied with synchronous multiple primary cancer was related to significantly impaired survival (p = 0.026). Further studies are therefore needed to determine the optimal treatments for patients with synchronous advanced esophageal and gastric cancers. It is possible that neoadjuvant chemotherapy and perioperative immunotherapy may prolong survival.

Besiede the stage and grades of cancer, which as risk factors for OS, we also found that lymph node metastasis and use of the jejunum for reconstruction were independent risk factors for OS. For patients with lymph node metastasis of cancers, the OS was especially poor. In contrast, Park et al. reported that the independent predictors of OS were advanced tumor stage (P = 0.008) and patient age (P = 0.009), but the type of esophageal conduit had no impact on early or late outcomes [21]. The effect of the type of conduit on late outcomes of these patients therefore requires more researches.

The existence of synchronous tumors of the esophagus and stomach is a not unusual, the radical surgical treatment could be carried out whenever possible. We anticipate that our findings will help surgeons to make better informed decisions and increase their confidence when attempting reconstruction using the colon or jejunum in patients who present with synchronous esophageal and gastric cancers. When total gastrectomy is inevitable, the optimal graft type should be selected carefully based on cancer location. Many factors, including operative time, surgical stress, preoperative complications, and cancer curability, must be considered when selecting the operative procedure. More studies are needed to examine the optimal treatment for patients with synchronous advanced esophageal and gastric cancers, and the possible benefits of neoadjuvant chemotherapy and immunotherapy on survival time may be investigated in future.

The most important limitation in this study was the nature of retrospective and single-institution. Increasing the sample size in future studies would have a more significant reference value for research and practice in the relevant field. Finally, the clinical outcomes maybe affected by the difference in age distribution between these two groups.

## Data Availability

The datasets used and/or analysed during the current study available from the corresponding author on reasonable request.
